# A Combination of a Vibrational Electromagnetic Energy Harvester and a Giant Magnetoimpedance (GMI) Sensor

**DOI:** 10.3390/s20071873

**Published:** 2020-03-27

**Authors:** Juan Jesús Beato-López, Isaac Royo-Silvestre, José María Algueta-Miguel, Cristina Gómez-Polo

**Affiliations:** 1 Departamento de Ciencias, Universidad Pública de Navarra, 31006 Pamplona, Spain; juanjesus.beato@unavarra.es (J.J.B.-L.); isaac.royo@unavarra.es (I.R.-S.); 2 Institute for Advanced Materials and Mathematics INAMAT2, Universidad Pública de Navarra, 31006 Pamplona, Spain; 3 Departamento de Ingeniería de Electricidad, Electrónica y Comunicación, Universidad Pública de Navarra, 31006 Pamplona, Spain; josemaria.algueta@unavarra.es; 4 Institute of Smart Cities, Universidad Pública de Navarra, 31006 Pamplona, Spain

**Keywords:** vibrational energy harvester, GMI effect, accelerometer, low frequency vibrating systems, amplitude

## Abstract

An energy harvesting device combined with a giant magnetoimpedance (GMI) sensor is presented to analyze low frequency vibrating systems. An electromagnetic harvester based on magnetic levitation is proposed for the electric power generation. The device is composed of two fixed permanent magnets at both ends of a cylindrical frame, a levitating magnet acting as inertial mass and a pick-up coil to collect the induced electromotive force. At the resonance frequency (10 Hz) a maximum electrical power of 1.4 mW at 0.5 *g* is generated. Moreover, an amorphous wire was employed as sensor nucleus for the design of a linear accelerometer prototype. The sensor is based on the GMI effect where the impedance changes occur as a consequence of the variations of the effective magnetic field due to an oscillating magnetic element. As a result of the magnet’s periodic motion, an amplitude modulated signal (AM) was obtained, its amplitude being proportional to mechanical vibration amplitude (or acceleration). The sensor’s response was examined for a simple ferrite magnet under vibration and compared with that obtained for the vibrational energy harvester. As a result of the small amplitudes of vibration, a linear sensor response was obtained that could be employed in the design of low cost and simple accelerometers.

## 1. Introduction

Energy harvesting can be defined as the green transformation of the environmental energy into electrical energy [1,2]; lately it has become a powerful field of research [1,3]. Different energy harvesting devices have been proposed in various technological applications; namely: biomedicine [4], structural health monitoring [5], remote monitoring systems [6], mobile phone [7], portable or wearable electronic devices [8,9], wireless systems [10] and biomedical implants [11]. The main reason for the expanded use in different technological areas is the possibility of feeding autonomous wireless sensor nodes under low power consumption conditions. In general, these nodes must be powered locally avoiding the extensive use of wires [1]. Although this constraint can be solved with the use of batteries, they must be replaced periodically [1,2], leading to service interruptions, environmental issues and high maintenance costs. In this sense, energy harvesters have been revealed as a way of extending the lifetimes of the sensor nodes [1] by increasing batteries’ durability or even via total replacement (self-powered devices).

Different types of energy harvesting devices can be found in terms of the source employed for the electrical energy generation: light, temperature, pressure gradients, radio frequency (RF) and motion or mechanical energy (vibrational harvesters) [12]. One of the most interesting fields is the vibrational harvesters, since vibrations are present in many technological sectors, such as industrial machinery, civil structures (bridges, buildings, etc.) and means of transport [13]. Aside from electrostatic [14], the most commonly employed transduction mechanisms are piezoelectric [11,15] and electromagnetic (based on Faraday’s Law) [16,17]. In general terms, they both can generate the same order of electrical power (milliwatts) [18] but they display different properties. Piezoelectric based harvesters generate a high output voltage; they have a structure without peripheral components, of a lower size and primed for ease of integration in microelectronic devices [1]. In contrast, due to their high impedance, they generate a low amplitude current output and suffer from depolarization [19], brittleness [20], low-temperature operation range [21] and stiffness (high natural frequency of oscillation) [22] requiring larger sizes for low vibration frequencies [23]. 

On the other hand, electromagnetic harvesters generate electric power as a result of the relative movement, with respect to a set of pick-up coils, of a permanent magnet (inertial mass) mechanically coupled to vibration. Overall, they generate low voltage outputs and are difficult to miniaturize due to the presence of coils together with the reported loss of efficiency of the energy conversion at reduced dimensions [18]. However, they exhibit higher amplitude output currents and a simple mechanical resonator structure that decreases the mechanical fatigue of the constituent components and allows a higher conversion efficiency at very low frequencies [22]. This is a relevant issue since, most natural vibrations have random frequencies [13] in a low range (1–10 Hz) with low accelerations (<9.8 m/s^2^) [24]; i.e., those related with human motion and machinery vibration [9]. Specifically, magnetic levitation electromagnetic harvesters based on permanent magnets have been revealed as a powerful tool for energy conversion in low frequency vibrating systems. In these vibrational harvesters, an effective magnetic restoring force is obtained on the magnet inertial mass through an arrangement of fixed magnets [25]. The fact that microtechnology is not required to manufacture magnet based transducers and mechanically coupled elements are missing, enable the production of low cost devices with reduced maintenance requirements and long lifetimes [9,26,27]. This simpler structure [27] together with the possibility to design the effective spring constant through the magnets’ configuration [9] justifies their employment in applications with strong constraints at low frequency of vibration. 

On the other hand, the analysis of vibrations itself can be of interest to prevent or identify damages in structures [28,29] or industrial machinery [30]. The characterization of these vibrations requires the determination of the amplitude of the motion (or acceleration) and its frequency which in general lies in the low frequency range [13]. This task is usually performed by piezoelectric commercial accelerometers attached to the vibrating element [23]. However, position sensors and accelerometers can be designed in many ways, involving different kinds of transducers and theoretical principles that produce sensors with different sizes, costs, sensitivities, bandwidths and precisions [31], including non-contact magnetic sensors [32,33]. For example, Reference [34,35] show the possibility of measuring vibrations using Hall sensors and permanent magnet markers, and in [31] the development of a self-powered vibration sensor based on magnets, springs and flexible coils is presented. However, linear accelerometers require a larger sensitivity to detect small variations of a magnetic field, as a result of the vibration of the permanent magnets. Hence, the giant magnetoimpedance (GMI) effect can be considered as a promising sensing principle for the design of linear magnetic accelerometers due to its large sensitivity. This effect consists in the great variations of the high frequency electrical impedance, *Z*, of a soft magnetic conductor under the action of an external magnetic field [36]. Concretely, in a previous work, a non-contact micrometric position sensor was proposed [37] constituting the basis of the presented magnetic linear accelerometer. In this device, Z changes due to the variation of the relative position of a permanent magnet generating the external magnetic field. 

In this work, a coupled device is proposed. It is composed of a magnetic levitation electromagnetic harvester and a linear GMI position sensor acting as an accelerometer. The harvester is comprised of two fixed magnets in the top and bottom of the frame and a mobile inertial mass that levitates between the magnets and inside of a coil. The designed harvester displays a resonance frequency of 10 Hz, characterized by maximum electromotive force and electrical power values. On the other side, the magnetic accelerometer is based on a soft magnetic amorphous wire (Co_66_Fe_12_Si_13_B_15_Cr_4_). The proposed accelerometer operation principle relies on the variations of the wire impedance under the variation of the effective magnetic field due to the magnets under vibration. This variable magnetic field leads to an amplitude modulated signal (AM) magnetoimpedance voltage, its amplitude being proportional to the mechanical vibration amplitude. The performance of the accelerometer is characterized by two magnetic systems (a single permanent magnet and the harvester) and the results are analyzed in terms of the relative displacement of the magnets elements. The proposed device not only can be used in the design of vibrational energy harvesters but it can also be used to build self-power vibrating sensing elements and highly sensitive magnetic accelerometers. Just from the perspective of the accelerometer, the proposed design based on a levitating magnet coupled to the vibration source and a GMI sensor, allows the development of low cost and miniaturized devices, broadening its potential applications.

## 2. Materials and Methods

### 2.1. Energy Harvester

**Figure 1:**
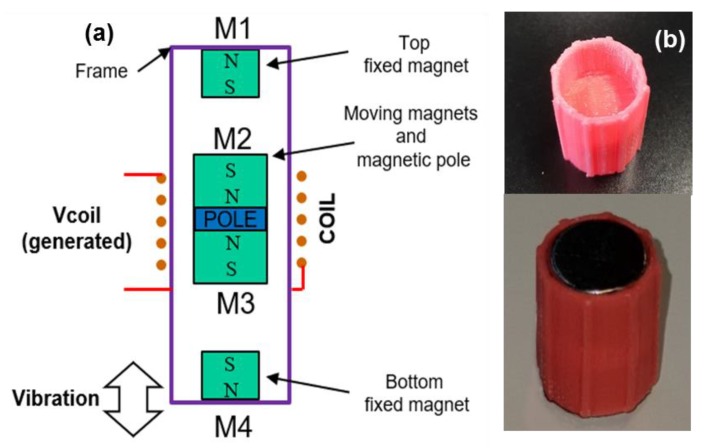
Harvester, (**a**) schematic diagram and (**b**) PLA part and final inertial mass with magnets.

The magnetic levitation based harvester is composed of a cylindrical frame, a coil and multiple permanent magnets (see Figure 1a) [38]. The coil and the top (M1) and bottom (M4) magnets are fixed to the frame, while the levitating inertial mass is composed of two magnets (M2, M3) which are glued to a spacer acting as a magnetic pole. The inertial mass is initially at rest in the equilibrium position, but it can move freely inside the frame. Under vibration, the magnetic forces generated by the fixed magnets leads to a virtual spring between the bottom frame and the inertial mass, forming a mass-spring-damper system with a characteristic resonance frequency [9]. The harvester is attached to a vibrating platform, characterized by an amplitude (*Y*_0_), while the inertial mass within the harvester vibrates under displacement *z*(*t*). The movement of the inertial mass (M2-M3) leads to changes in the magnetic flux through the coil, generating an electromotive force (*emf*), *ε*.

To reduce the mechanical friction between the inertial mass and the frame, a 3D printed PLA (polylactic acid) plastic housing with guide rails was designed to encase and guide the inertial mass. Figure 1b shows the designed PLA frame and the final stator with the fixed magnets. Table 1 summarizes the main characteristics of the designed prototype.

The harvester was placed on a shaker (LDS V201) that was excited with a function generator (AFG 310, Sony Tektronix) and an *AC* linear power amplifier (LDS, LPA100 Brüel and Kjaer). A low frequency sinusoidal signal was employed, with frequency ranging from 10 to 40 Hz. The acceleration of the vibrating motion was registered by a commercial accelerometer with negligible mass (PCD, 352C33). The output voltage of the accelerometer (a quasi-sinusoidal signal with 9.8m⁄s^2^ corresponding to 100 mV peak to peak) and the electromotive force generated by harvester (*ε*) were acquired with an oscilloscope (Tektronix MDO 3024).

### 2.2. GMI Magnetic Field Sensor

**Figure 2:**
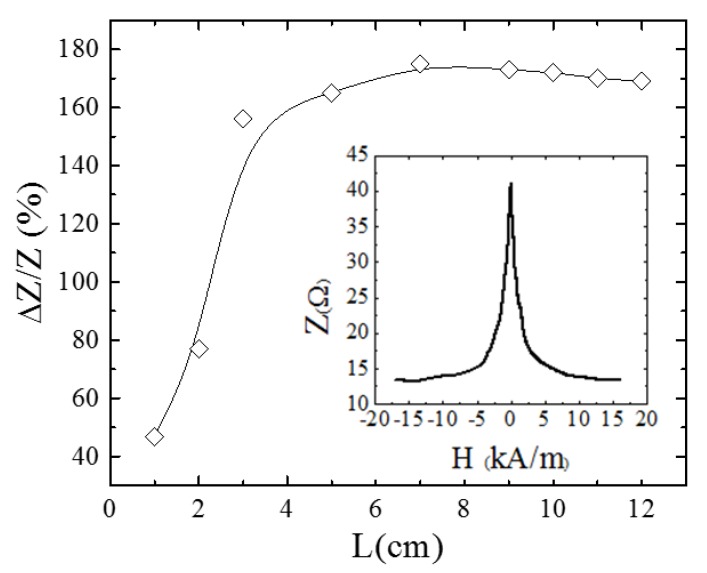
Giant magnetoimpedance (GMI) ratio $\frac{\Delta Z}{Z}\;\left(\%\right)$ as a function of the wire’s length. Inset: Z versus the applied magnetic field, *H*, for L = 3 cm, (*f_GMI_* = 300 kHz and *I_pp_* = 19 mA).

The sensor nucleus was an amorphous wire with nominal composition Co_66_Fe_12_Si_13_B_15_Cr_4_ and a mean diameter of 90 µm obtained by in-rotating water quenching technique [39]. The first step was to determine the optimal conditions leading to the maximum GMI effect. For that, a 3 cm in length wire was excited with a function generator (Standford Research System DS 345) with a sinusoidal *AC* signal with frequency *f_GMI_* and peak to peak current amplitude, *I_pp_* under a voltage divider configuration. The GMI peak-to-peak voltage, *V*, ($V=ZI_{pp}$, *Z* electrical impedance) was measured by a Hewlett Packard 34401A multimeter. In this configuration, the GMI ratio, defined as the maximum relative variation in impedance ($\frac{\Delta Z}{Z}\left(\%\right)=\frac{Z\left(0\right)-Z\left(H_{MAX}\right)}{Z\left(H_{MAX}\right)}\times 100$; *Z*(0): impedance at zero applied magnetic field (*H* = 0); *Z*(*H_MAX_*): impedance at maximum applied magnetic field (*H_MAX_* = 16 kA/m )) being the *DC* axial *H* field generated by a home-made solenoid. The whole system was controlled by LABVIEW 2014. Optimal conditions (maximum GMI ratios) were found at *f_GMI_* = 300 kHz and *I_pp_* = 19 mA. Under these optimum conditions, the effect of the sample length on the GMI ratio was analyzed. As Figure 2 displays, maximum GMI ratios around 170% were found for sample lengths *L* ≥ 7 cm. Nevertheless, *L* = 3 cm was chosen as a compromise among sensitivity and applicability in the final designed prototype. The inset of Figure 2 shows the evolution of the impedance, Z, as a function of the applied magnetic field, *H*, for this sample length under the selected optimum exciting conditions. 

**Figure 3:**
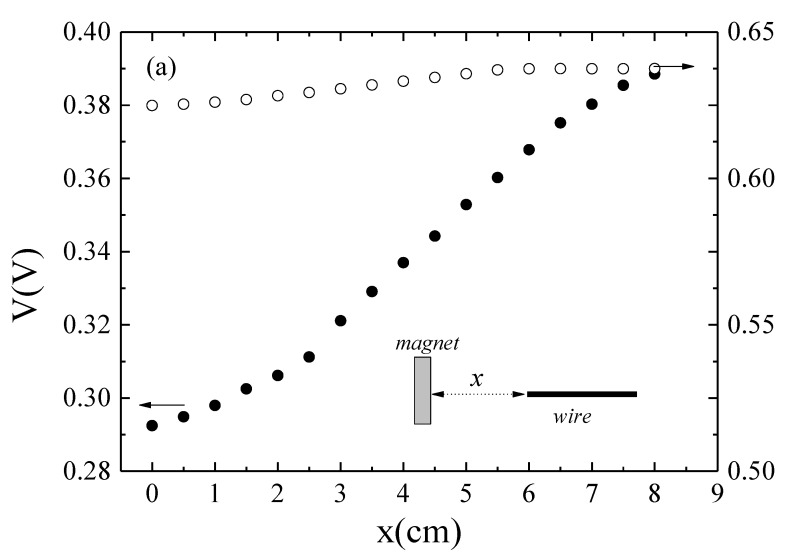

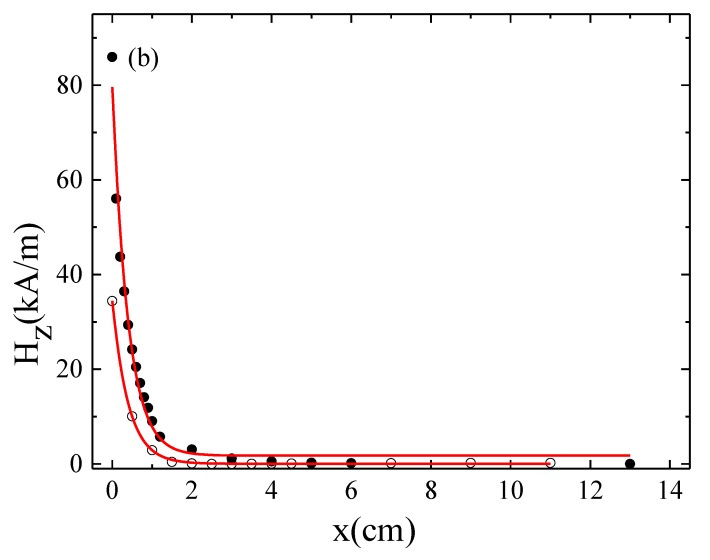
(**a**) GMI voltage, *V*, and (**b**) magnetic field, *H_z_*, as a function of the relative distance, *x*, between the magnet and the GMI sensor (wire) for (●) the ferrite magnet and (o) the energy harvester. Inset in (**a**): schematic of the experimental configuration. The solid line in (**b**) represents the theoretical estimated field according to Equations (A2) and (A3) (Appendix A).

Subsequently, the response of the sensor to a non-homogeneous *DC* magnetic field, *H_z_*, was characterized employing a ferrite rectangular magnet (1.0 × 1.7 × 0.5 cm), by evaluating the changes of *Z* as a function of the relative distance, *x*, between the magnet top surface and the wire. Starting from mutual contact (*x* = 0) the sensor voltage, V, was measured with a step of *x* = 0.5 cm (see inset of Figure 3a). Similarly, the sensor response was characterized employing the magnetic field generated by the energy harvester. In this case, the wire was placed along the axis of the cylindrical frame at a distance *x* from the upper part of the frame. Figure 3a shows the comparative response in both configurations: rectangular magnet (black circles) and the energy harvester with the inertial mass at the equilibrium position (white circles). 

Analogously to the voltage characterization, the magnetic field was evaluated through the measurement of *H_z_* versus *x* in both configurations employing a Lakeshore 425 gaussmeter (see Figure 3b). The solid lines in Figure 3b are the estimation of the magnetic field employing the expressions for magnets based on [40] (see Appendix A). Despite the higher strength of the magnetic field generated by the individual NdFeB magnets employed in the harvester, the effective *H_z_* along *x* for the harvester is slightly lower than the measured values of the rectangular magnet. This behavior is clearly reflected in the evolution of *V* under both magnet configurations, achieving the highest sensor voltage variations under the highest magnetic field strength of the ferrite magnet. 

### 2.3. Vibration Measurement

**Figure 4:**
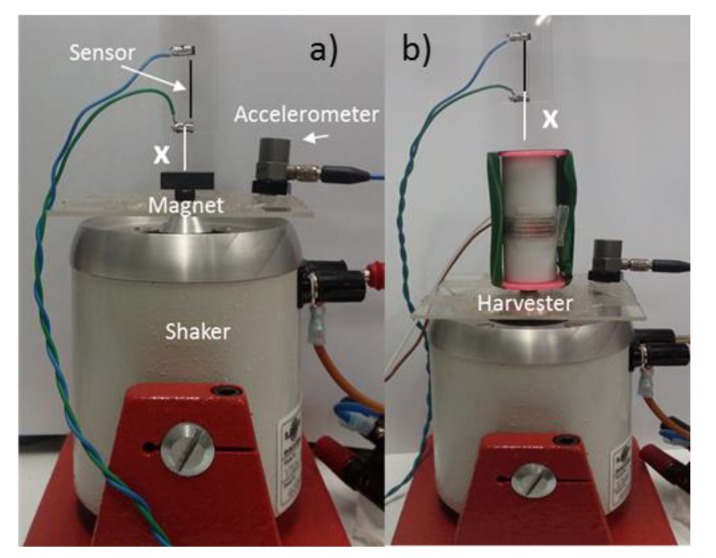
Experimental setup employed in the vibrating system (shaker): the accelerometer and the GMI sensor, (**a**) the ferrite magnet; (**b**) the harvester.

After the initial characterization of the GMI wire, the performance of the sensor was checked while the system was subject to low frequency vibrations. Figure 4 shows the experimental set-up for the two analyzed magnetic systems: (a) ferrite magnet and (b) energy harvester. As can be seen, the magnetic element was attached to the vibrating platform (shaker) describing a harmonic motion that modified the relative distance, *x*, to the wire. Initially, the GMI sensor was placed at rest (fixed to the ground) at a distance *x* = 1.5 cm above of the top side of the ferrite magnet or the upper part of the harvester frame (the distance from the harvester frame to the top side of the M1 magnet was *λ* = 0.3 cm, see Appendix A). Note that in this case the magnetic field of the harvester acting on the sensor is the superposition of the magnetic field generated by the movement of the fixed magnets (mainly M1) and the relative displacement of the inertial mass (M2-M3). Afterward, the GMI sensor was fixed to the shaker platform simultaneously vibrating with the harvester (*x* constant); this configuration allowed for the characterization of the magnetic field changes due to the inertial mass motion.

**Figure 5:**
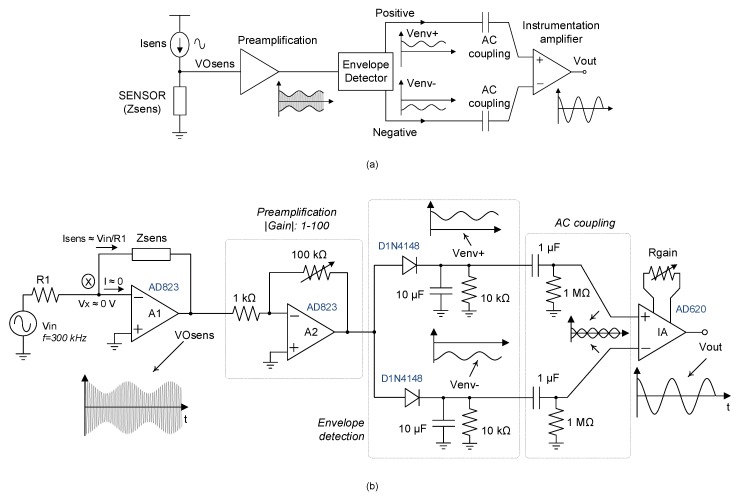
Analog interface for the vibration sensor: (**a**) block diagram and (**b**) circuit implementation.

For all cases, as a consequence of the periodic changes of the magnetic field on the wire, an *AM* modulated output voltage signal was obtained in the GMI sensor, *V_0sens_* (see Figure 5a,b). The closest position between the sensor and the magnet elements (highest *H_z_* acting on the wire; see Figure 3) corresponds to a minimum value of the impedance (see inset of Figure 2a) and thus to a minimum sensor voltage and vice versa. Accordingly, the modulation depth of *V_0sens_* enables the characterization of the amplitude of oscillation. A homemade analog electronic interface was designed for the signal deconvolution. 

The general scheme of the design analog interface is shown in Figure 5 where (a) represents the underlying idea and (b) is its practical implementation. As can be seen, the wire sensor is excited with a sinusoidal signal, *V_in_* (*f_GMI_* = 300 kHz) and the resistor value *R_1_* was properly chosen to operate under optimal GMI conditions (*I_sens_* = *I_pp_* = 19 mA). Due to the periodic changes in *H_z_*, the amplitude of the enveloping signal, *V_0sens_*, (*V_0sens_* = −*I_sens_*.*Z*) depends on the extreme values (minimum and maximum) of *Z* during vibration, or, in other words, on the difference of maximum and minimum *H_z_* acting on the sensor. To detect the modulation depth of *V_0sens_* signal, a double passive envelope detector (positive and negative) was employed [41]. The resulting signals *V_env+_* and *V_env-_* were *AC* coupled to an instrumentation amplifier AD620 in order to obtain the signal *V_out_*, which was proportional to the differential envelope of *V_0sens_*.

The amplitude of *V_out_* was measured using the oscilloscope for several vibration amplitudes of the vibrating platform at different frequencies of 10, 30 and 40 Hz. No signal *V_out_* was detected when the relative distance between the vibrating magnetic element and GMI sensor remained constant. 

## 3. Results

**Figure 6:**
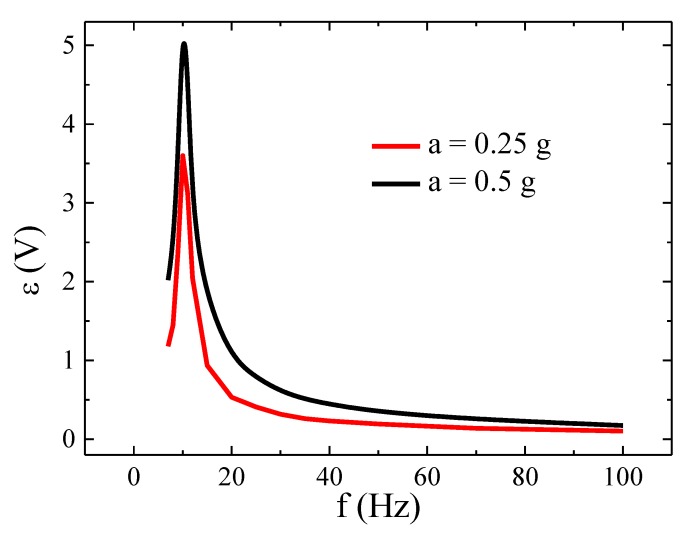
Induced *emf*, *ε*, in the harvester as a function of vibrating frequency for the accelerations: *a* = 0.5 g and *a* = 0.25 g.

**Figure 7:**
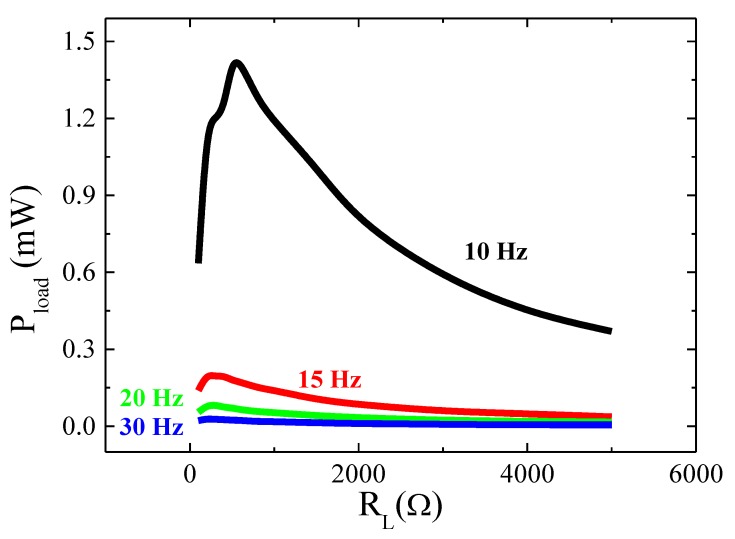
Electrical power, *P_load_*, versus the resistive load, *R_L_* for an acceleration of *a* = 0.5 g.

Firstly, the energy harvester was characterized and the induced *emf*, *ε*, evaluated as a function of the vibration frequency, *f*, in an open circuit for the accelerations of *a* = 0.25 and 0.5 g (peak value, measured through the commercial accelerometer). As Figure 6 shows a resonance frequency *f_res_* ≈ 10 Hz was obtained, characterized by a maximum value in *ε*. Afterward, the electrical power, $P_{load}$, generated by the energy harvester was analyzed as a function of the external resistive load, *R_l_*, connected to the coil for different frequencies and at a constant vibration acceleration amplitude *a* = 0.5 g. $P_{load}$ values were estimated through the expression, $P_{load}=\frac{Vload^{2}}{R_{L}}$, where *V_load_* is the voltage measured in the load resistance. As can be seen in Figure 7, a maximum in *P_load_* was observed for *R_L_* = 500 Ω at the resonance frequency. A sharp decrease in *P_load_* was obtained for higher vibration frequencies irrespectively of the load resistance. Similar values (≈mW) of the generated electrical power have been reported in the literature for equivalent vibrational harvesters [18]. As an example, values of 1.53 mW (*f* = 20 Hz and *a* = 0.4 g) and 0.74 mW (*f* = 16 Hz and *a* = 0.4 g) were obtained in [42,43] but at much higher loads, 200 and 55 kΩ respectively.

**Figure 8:**
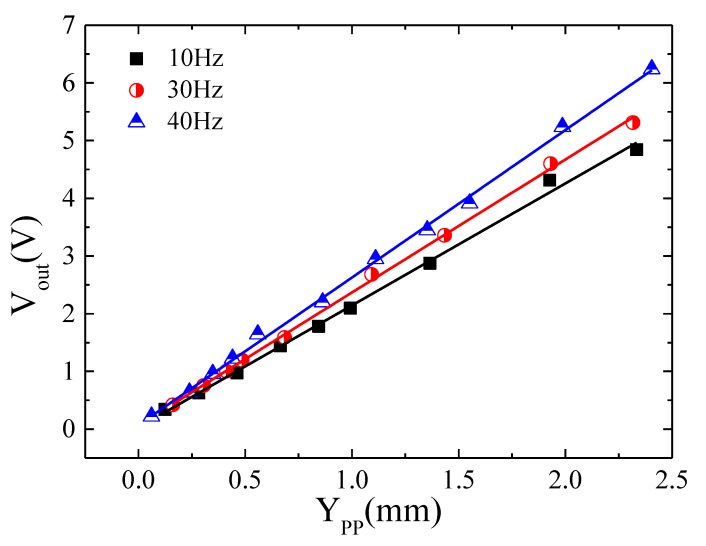
Peak to peak sensor output signal *V_out_*, versus peak to peak amplitude, *Y_pp_*, of vibration of the rectangular permanent magnet.

Then, the characterization of the vibration by means of the GMI sensor was performed. The first step was to examine the GMI sensor response in the simplest system; that is, the rectangular magnet attached to the vibration platform (see Figure 4a). In this case, the amplitude of vibration of the magnetic system, *Z*_0_, is equal to the peak amplitude of the vibrating platform, *Y*_0_. This amplitude value can be obtained from the vibration angular frequency ω=2πf, and the acceleration amplitude, *a*, measured by the commercial accelerometer *Y*_0_ $=\frac{a}{\omega^{2}}$. Figure 8 shows the peak to peak output signal of the sensor, *V_out_*, as a function of *Y_pp_* = 2 *Y*_0_ for different vibration frequencies (*f* = 10, 30 and 40 Hz). A linear response was obtained with sensitivity, *G*, (slope of *V_out_* versus *Y_pp_*), nearly independent of the vibrational frequency: *G* = 2.11 ± 0.05 (*f* = 10 Hz), 2.30 ± 0.03 (*f* = 30 Hz) and 2.56 ± 0.03 (*f* = 40 Hz) V/mm. These results clearly show the suitability of the proposed GMI sensor to characterize low frequency mechanical vibrations, through the determination of the displacement and acceleration amplitude experienced by the vibrating system. It is relevant to note that, as expected from the designed analog interface, the resulting *V_out_* signal had the same frequency as the vibration.

**Figure 9:**
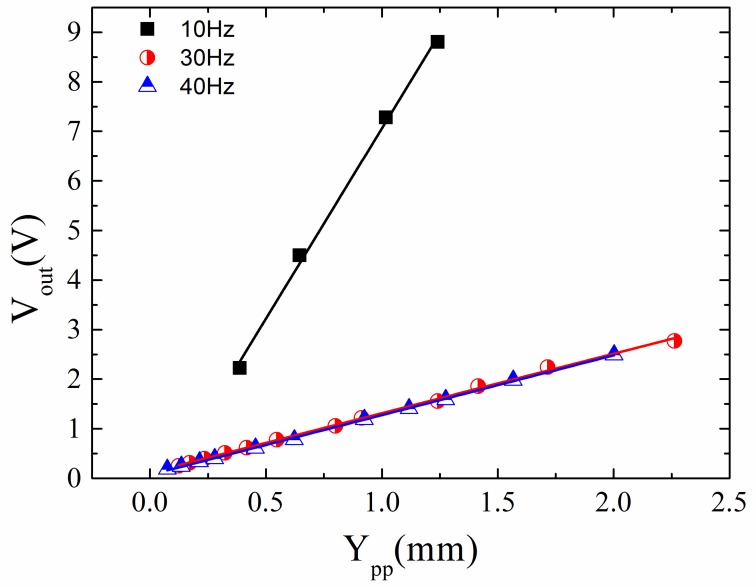
Sensor output signal *V_out_*, versus amplitude, *Y_pp_*, of vibration of the energy harvester (sensor at rest with respect to the vibrating platform).

**Figure 10:**
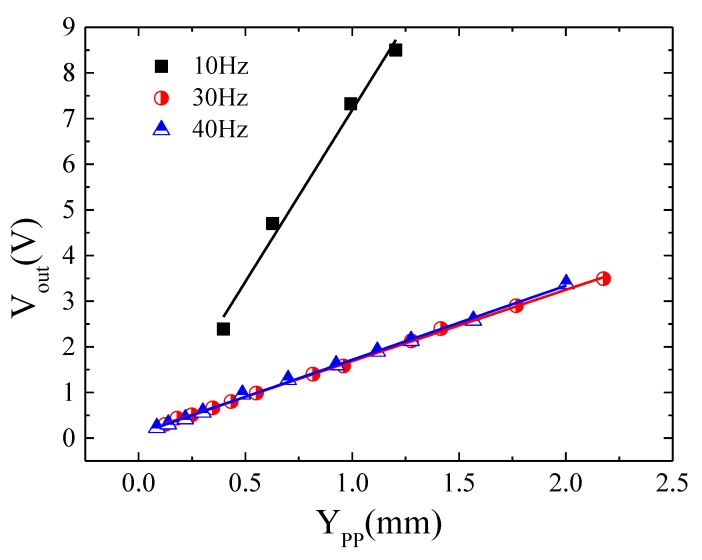
Sensor output signal *V_out_*, versus amplitude, *Y_pp_*, of vibration of the energy harvester (sensor solidary vibrating with the vibrating platform).

Comparatively, Figure 9 and Figure 10 show *V_out_* versus *Y_pp_* for the harvester in the two cases, keeping the GMI sensor at rest and fixed to the vibrating platform of the shaker, respectively (see Figure 4). It is important to note that while in the first analyzed configuration, the distance *x* between the harvester frame and GMI is time dependent, in the second case it is constant, so only the relative movement of the magnetic inertial mass is registered. In both cases, a linear response is obtained for the three analyzed vibration frequencies with maximum sensitivity at the resonance frequency: *G* = 7.70 ± 0.03 (*f* = 10 Hz) for the sensor at rest and *G* = 7.5 ± 0.6 (*f* = 10 Hz) when it is attached to the vibration platform. Off-resonance, the sensitivity values were also similar for both configurations: *G* = 1.19 ± 0.02 (*f* = 30 Hz), 1.20 ± 0.01 (*f* = 40 Hz) V/mm for the sensor at rest and *G* = 1.56 ± 0.01 (*f* = 30 Hz), 1.62 ± 0.02 (*f* = 40 Hz) V/mm for the sensor vibrating with the vibrating platform. The similarity of the *G* values under both configurations indicates that the magnet inertial mass (M2-M3) mainly dominates the changes of the magnetic field outside the harvester. Nevertheless, off-resonance, slightly lower sensitivity values were registered when the sensor was fixed at rest. This fact can be explained in terms of the slightly different magnetic field acting on the sensor in each configuration. When the sensor vibrated with the vibration platform, the magnetic field acting on the wire is only due to the inertial mass. However, when the sensor is fixed at rest, the contribution of the magnetic field of the upper magnet in the frame should be taken into account (changes in x). Since both magnetic fields have opposite senses (see Figure 1a), the net strength of the effective magnetic field is slightly lower intense for the same amplitude of vibration *Y_pp_*, leading to lower output signal *V_out_*. This effect at resonance is negligible because of the higher amplitude of vibration described by inertial mass.

Besides, the sensitivities displayed by both piezoelectric (50 mV/g; considering the peak value of acceleration) and magnetic accelerometers were compared. For the GMI based accelerometer, the sensitivities in terms of the peak acceleration can be calculated through the expression, a=Yoω2=Ypp2ω2, resulting in a frequency dependent behavior. In consequence, only the less favorable case of sensitivity was compared. This case corresponds to the larger frequency of vibration, f = 2πω = 40 Hz and when the sensor was at rest (see Figure 9). Under these conditions, an almost double sensitivity (95 mV/g) was obtained for the magnetic accelerometer. For lower frequencies, the difference is even larger. Finally, in addition to the larger sensitivity obtained, it is relevant to remark that the estimation of the acceleration has been performed remotely, without the necessity for simultaneous oscillation with the vibrating surface. This fact potentially paves the way for the design of low cost and consumption accelerometers, although a study of the miniaturization of the prototype components is required. 

## 4. Discussion

In general terms, the harvester is a non-linear device [9], but, assuming a sinusoidal excitation *y*(*t*) = *Y*_0_ cos(*ωt*), its behavior can be described by a set of simplified formula, with negligible static friction and constant resonant frequency. Under this assumption, the inertial mass displacement, *z*(*t*), can be expressed as a function of the input vibration amplitude, *Y*_0_, through the expression given by [44]:

(1) $$z\left(t\right)=Z_{0}\cos\left(\omega t+\theta \right)$$

(2) $$Z_{0}=Y_{0}\frac{{\left(\frac{\omega }{\omega_{n}}\right)}^{2}}{\sqrt{{\left[1-{\left(\frac{\omega }{\omega_{n}}\right)}^{2}\right]}^{2}+{\left(\frac{2\zeta \omega }{\omega_{n}}\right)}^{2}}}$$

where *ω_n_* is the angular resonant frequency (*ω_n_* = 2π*f*_res_), *ζ* the damping ratio and *θ* the phase between vibration and inertial mass displacement; $\theta =-\tan^{-1}\frac{2\zeta \frac{w}{w_{n}}}{1-{\left(\frac{w}{w_{n}}\right)}^{2}}$ (at resonance *θ* = −π/2 radians). In this simplified model the damping ratio takes into account both mechanical friction and electrical phenomena [45] and thus *ζ* = *ζ_p_* + *ζ_e_* (*ζ_p_* is the parasitical damping ratio due to mechanical friction; *ζ_e_* electromagnetic damping coefficient) [46]. While *ζ_p_* is constant for a given device, *ζ_e_* varies with the load resistor and equals zero for open circuit coil [45]. Thus, maximum amplitudes, *Z*_0_, of the inertial mass displacement are achieved at *f* ≈ *f_res_* as the electromotive force induced in the harvester reflects (*f_res_* ≈ 10 Hz, see Figure 6). 

Concerning the GMI sensor response, if the measurement configuration lacks a magnetic spring, the amplitude of vibration of the magnetic element, *Z*_0_, (rectangular magnet in this case) coincides with the amplitude of the input vibration to characterize, *Y*_0_ (*Z_0_* = *Y*_0_). Under these circumstances, the sensor voltage *V_out_*, and thus the sensibility, *G*, should be frequency independent, as experimentally confirmed (see Figure 8):

(3) $$V_{out}=G(2Y_{0})$$

where *Y_pp_* = 2*Y*_0_ (amplitude employed in the experimental characterization). Such a linear behavior should be interpreted as the result of the small variations of the relative distance *x* between the permanent magnet and the GMI sensor during vibration. In fact, displacements in the millimeter range around the initial equilibrium position (*x* ≈ 1. 5 cm) lead to a linear magnetoimpedance response (see Figure 3). 

On the other hand, under magnetic spring configuration (harvester), the inertial mass dominates the magnetic field strength at the measuring point and its displacement *Z*_0_ is related to the shaker vibration, *Y*_0_ by Equation (2). Accordingly, the output voltage can be expressed in terms of *Y*_0_ as:

(4) $$V_{out}=G\cdot Z_{0}=G\frac{{\left(\frac{\omega }{\omega_{n}}\right)}^{2}}{\sqrt{{\left[1-{\left(\frac{\omega }{\omega_{n}}\right)}^{2}\right]}^{2}+{\left(\frac{2\zeta \omega }{\omega_{n}}\right)}^{2}}}Y_{0}$$

Therefore, a marked frequency dependent behavior is obtained under the harvester configuration (see Figure 9 and Figure 10), where, for a given *Y*_0_, maximum output voltages are achieved at *f* ≈ *f_res_* as a consequence of the minimum value of the denominator in eq.4. Moreover, as vibrating frequency increases with respect to *f_res_*, the denominator increases, leading to a decrease in *V_out_* value. In fact, the effect of the vibrating frequency plus the damping parameter, ζ, facilitates a rapid decrease in *V_out_* observing a diminution close to one order of magnitude in the achieved sensitivity (from 7.5 at 10 Hz to 1.56 V/mm at 30 Hz, sensor vibrating with the platform). Almost no difference in the sensor response was found when compared with the highest analyzed vibrating frequency of 40 Hz (1.60 V/mm). 

**Figure 11:**
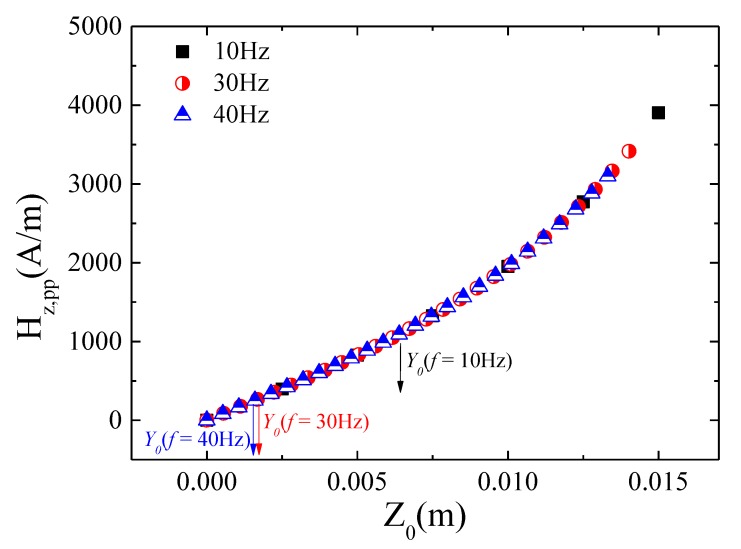
Theoretical magnetic field amplitude, *H_zpp_*, as a function of the harvester inertial mass displacement amplitude, *Z*_0_. The maximum experimental harvester amplitudes of vibration, *Y*_0_ = 1.25 mm, are indicated.

Furthermore, a linear relationship was also found in *V_out_* versus the amplitude of vibration for the harvester under both analyzed situations (sensor at rest or fixed to the vibrating platform; see Figure 9 and Figure 10). This linear response supports again the linear relationship between the small vibration amplitudes (in this case of the inertial mass) and the variation of the effective magnetic field acting on the GMI sensor. Figure 11 shows the estimated magnetic field amplitude, *H_zpp_*; namely, the effective peak to peak value of the magnetic field (*H_max_* − *H_min_*)—see Appendix A—as a function of the inertial mass displacement amplitude, *Z_0_*. As described in Appendix A, the estimation of *H_zpp_* is performed employing the analytical expression for the magnetic field of a cylindrical magnet (Equation (A1)) and the superposition principle of the magnetic field generated by the set of magnets (M1, M2, M3 and M4) in the harvesters (Equation (A4)), assuming only the initial magnet under vibration. Additionally, the displacement amplitude values (*Z*_0_) are obtained through Equation (4), considering ζ = *ζ_p_* = 0.1 (open coil). So, as it is shown in Figure 11, even the maximum displacements of $Y_{0}=\frac{Y_{pp}}{2}=1.25\;mm$ (see also Figure S1) take place in a region where *H_zpp_* depends linearly on *Z_0_*, justifying the linear experimental response of the GMI sensor under the vibration of the inertial mass of the harvester. 

In conclusion, the system has demonstrated its capability for first generating electrical energy from low frequency environmental vibrations, and finally, characterization. Nevertheless, several issues have to be addressed before a final and commercial prototype. The response of the magnetic linear accelerometer has been tested under sinusoidal excitation. A deeper analysis must be performed under a time-variable vibration source where no well-defined input signal is produced. Besides, the electric energy generation rapidly decreases off-resonance. So, a broadening of the frequency bandwidth interval where resonance takes place is necessary to optimize the energy generated by the system. Once these two intermediate goals are achieved the final step can be faced; that is, to design an electronics capable of transmitting the harvested energy for the feeding of the GMI accelerometer, and so, achieving an autonomous self-powered device.

## 5. Conclusions

A combined system for the generation of the electric energy from environmental vibration and its simultaneous characterization, in terms of amplitude and/or acceleration, has been designed.

The magnetic levitation harvester is comprised of two fixed NdFeB magnets, in the top and bottom of the cylindrical frame, and a mobile inertial mass also composed of NdFeB magnets that levitates between the magnets and inside of a coil. At the characteristic resonance (*f* ≈ 10 Hz) it generates an electromotive force around 5 V in open circuit and an electrical power of 1.4 mW with a resistance load of 500 Ω, enabling its application for harvesting energy purposes. As expected, a fast decrease of both magnitudes is observed at off-resonance. 

With respect to the proposed magnetic linear accelerometer, a soft magnetic amorphous wire was employed as a sensor element, characterized by maximum impedance changes close to 160% under saturating magnetic *DC* fields. As a result of the magnet’s periodic motion, an *AM* modulated signal was obtained, its amplitude being proportional to mechanical vibration amplitude (or acceleration). The device has demonstrated its capability to characterize low frequency vibrational motion (*f* ≈ 10 Hz), both in the magnetic harvester and in the simplest case with a single permanent ferrite magnet. The sensor response (output voltage) displays in all the analyzed cases a linear dependence on the amplitude of vibration. Such a linear relationship is explained in terms of the small variations of the magnetic field in the sensor due to the low amplitude vibrations. A nearly frequency independent sensitivity, *G*, was obtained for the simplest analyzed system (ferrite permanent magnet). However, a frequency dependent behavior was found for the harvester as a consequence of the resonant response, with the highest *G* at the resonance frequency with respect to the off-resonance state. Nevertheless, it can be concluded that the proposed sensor exhibits enough sensitivity for the characterization of low frequency vibrations (*f* ≤ 40 Hz) irrespectively of the sensor position (at rest or vibrating with the harvester) concerning the vibration system. In fact, the possibility to employ it without mechanical contact with the vibrating system represents a remarkable advantage with respect to commercial piezoelectric accelerometers that are required to oscillate simultaneously with the vibrating surface. Nevertheless, when the GMI accelerometer needs to be located attached to the vibrating platform due to experimental restrictions, it shows a competitive response in comparison with commercial piezoelectric accelerometers. The availability of these two configurations broadens the application fields of the sensor. In fact, the proposed design based on a levitating magnet coupled to the vibration source and a GMI sensor can be employed in the design of low cost and miniaturized accelerometers. However, it should be kept in mind that the main application of the proposed device is the design of build self-autonomous devices capable of generating electrical power and simultaneously being able to remotely monitor vibrations. 

## Figures and Tables

**Table 1:** Characteristics of the designed energy harvester prototype: *ζ_p_* is the parasitical damping ratio, *Ø* is the diameter (inner/outer), *h* the height, *y* is the vertical position of each element center considering *y* = 0 as the middle of the Teflon tube, *N* is the number of turns of the coil and *R is* its resistance.

Element	Value
*Harvester*	Volume 12.7 cm^3^ *ζ_p_* = 0.1
*Frame*	*Ø* = 15/17 mm, *h* = 62 mm Rigid Teflon
*Inertial mass*	14.5 g
*Fixed magnets (M1 and M4)*	*Ø* = 10 mm, *h* = 1 mm *y* = ±27.5 mm NdFeB N35
*Moving magnets (M2 and M3)*	*Ø* = 12 mm, *h* = 8 mm NdFeB N45
*Magnetic pole*	*Ø* = 12 mm, *h* = 5 mm Non-magnetic (plastic)
*Coil*	*Ø* = 17/18 mm, *h* = 6 mm *y* = −3.5 mm *N* = 1000 turns copper wire *Ø* = 70 µm *R* = 250 Ω

**Figure A1:**
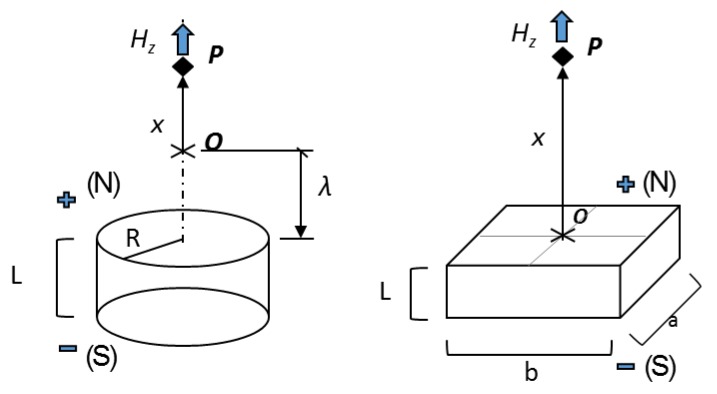
Analytical general model for axial field calculation of (left) a cylinder magnet with axial magnetization and (right) a rectangular magnet.

## Supplementary Materials

The following are available online at https://www.mdpi.com/1424-8220/20/7/1873/s1. **Figure S1:** Functional dependence between, *H_zpp_*, and $Y_{0}$.

## Appendix A

In order to study the theoretical behavior of the whole sensor system using analytical expressions, a moderate complexity method to calculate the magnetic field of a single permanent magnet by [40] was chosen; then a set of formulae for multiple moving magnets was derived.

The formulae in [40] are refactored and generalized for, resulting in the following equations (see Figure A1). It should be noted that these expressions yield the magnitude of the axial magnetic field in the axis (absolute value).

(A1) $$H_{z}=\frac{M}{2}\cdot \left(\frac{x+\lambda +L}{\sqrt{{\left(x+\lambda +L\right)}^{2}+R^{2}}}-\frac{x+\lambda }{\sqrt{{\left(x+\lambda \right)}^{2}+R^{2}}}\right),\;\mathrm{cylindrical}\;\mathrm{magnet}$$

(A2) $$H_{z}=\frac{M}{\pi }\cdot \left[\arctan\left(\frac{ab}{4x\sqrt{\frac{a^{2}}{4}+\frac{b^{2}}{4}+x^{2}}}\right)-\arctan\left(\frac{ab}{4\left(x+L\right)\sqrt{\frac{a^{2}}{4}+\frac{b^{2}}{4}+{\left(x+L\right)}^{2}}}\right)\right],\;\mathrm{rectangular}\;\mathrm{prism}$$

where *M* is the volume magnetization; *R* the radius of the cylindrical magnet; *L* the magnet length in the *z*-axis; *a, b* are the side dimensions of the rectangular magnet; *x* the point O to the sensor position P (top surface of the rectangular magnet and frame for the harvester); and *λ* the distance from the harvester upper magnet face to point O.

For the rectangular ferrite magnet, *M* = 350 kA/m was used for the estimation. The harvester uses two kinds of NdFeB magnets, *M* = 868 kA/m for N35 magnets (M1 and M4 in Figure 1) and *M* = 923 kA/m for N45 magnets (M2 and M3 in Figure 1).

The magnetic field of the harvester can be found by the superposition principle, adding the magnetic fields created by each aligned magnet using the proper sign. Thus, if *H*_1_, *H*_2_, *H*_3_ and *H*_4_ are the fields of magnets M1, M2, M3 and M4, respectively, then the net magnetic field will be

*H_harv_* = *H*_1_ − *H*_2_ + *H*_3_ − *H*_4_ (A3)

Under movement, the position variables should be replaced by time dependent functions to obtain *H_harv_*(*t*). As an example, for the case of the sensor vibrating attached to the vibrating platform, the relative distances for magnets M2 and M3 are time functions so *λ*_2_ = *λ*_2*r*_ − *z*(*t*) and *λ*_3_ = *λ*_3*r*_ − *z*(*t*) being *λ_ir_* the distance at rest of each magnet to point O and *z(t)* the movement of the inertial mass (Equation (1)). Thus, the peak values of the magnetic field can be determined analytically: *H_max_* for *z*(*t*) = *Z*_0_ and *H_min_* for *z*(*t*) = −*Z*_0_. Then, the peak to peak value of the magnetic field waveform is *H_pp_* = *H_max_* − *H_min_*. Substituting and simplifying to *x* = 0, the final equation is:

(A4) $$H_{z,pp}=\frac{M_{2}}{2}\cdot \left(\frac{\lambda_{2r}+Z_{0}+L_{2}}{\sqrt{{\left(\lambda_{2r}+Z_{0}+L_{2}\right)}^{2}+R_{2}^{2}}}-\frac{\lambda_{2r}-Z_{0}+L_{2}}{\sqrt{{\left(\lambda_{2r}-Z_{0}+L_{2}\right)}^{2}+R_{2}^{2}}}+\frac{\lambda_{2r}-Z_{0}}{\sqrt{{\left(\lambda_{2r}-Z_{0}\right)}^{2}+R_{2}^{2}}}-\frac{\lambda_{2r}+Z_{0}}{\sqrt{{\left(\lambda_{2r}+Z_{0}\right)}^{2}+R_{2}^{2}}}\right)\;-\frac{M_{3}}{2}\cdot \left(\frac{\lambda_{3r}+Z_{0}+L_{3}}{\sqrt{{\left(\lambda_{3r}+Z_{0}+L_{3}\right)}^{2}+R_{3}^{2}}}-\frac{\lambda_{3r}-Z_{0}+L_{3}}{\sqrt{{\left(\lambda_{3r}-Z_{0}+L_{3}\right)}^{2}+R_{3}^{2}}}+\frac{\lambda_{3r}-Z_{0}}{\sqrt{{\left(\lambda_{3r}-Z_{0}\right)}^{2}+R_{3}^{2}}}-\frac{\lambda_{3r}+Z_{0}}{\sqrt{{\left(\lambda_{3r}+Z_{0}\right)}^{2}+R_{3}^{2}}}\right)$$
