# Supplementary material for: A Combination of a Vibrational Electromagnetic Energy Harvester and a Giant Magnetoimpedance (GMI) Sensor
Source: Sensors (Basel). 2020 Mar 27;20(7):1873. doi: 10.3390/s20071873 (PMC7180720; doi:10.3390/s20071873)
Supplement: Supplementary file 1 [file sensors-20-01873-s001.pdf]

Supplementary material)

Vibrational electromagnetic energy harvester and Giant Magnetoimpedance (GMI) sensor combined device

J. J. Beato-López[1, 2], I. Royo-Silvestre[1], J. M. Algueta-Miguel[3,4], C. Gómez Polo[1,2*]

[1] Dpto. de Ciencias, Universidad Pública de Navarra. 31006 Pamplona, Spain; juanjesus.beato@unavarra.es, isaac.royo@ unavarra.es, gpolo@unavarra.es

[2] Institute for Advanced Materials and Mathematics, INAMAT[2], Universidad Pública de Navarra. 31006 Pamplona, Spain; juanjesus.beato@unavarra.es, gpolo@unavarra.es

[3] Dpto. de Ingeniería de Electricidad, Electrónica y Comunicación, Universidad Pública de Navarra, 31006 Pamplona, Spain; josemaria.algueta@unavarra.es

[4] Institute of Smart Cities, Universidad Pública de Navarra, 31006 Pamplona, Spain, josemaria.algueta@unavarra.es.

* gpolo@unavarra.es; Tel.: +34-948-169-576.

Received: date; Accepted: date; Published: date

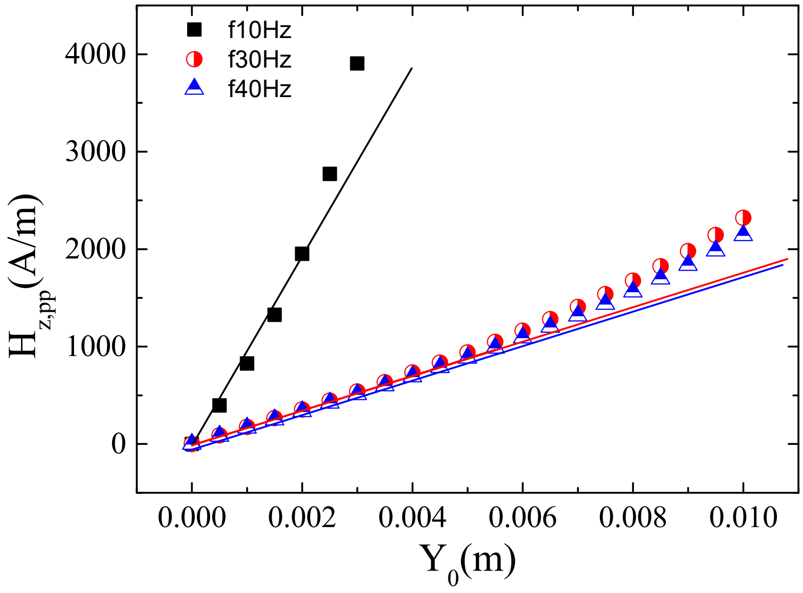

Figure S1: Functional dependence between, H_{zpp}, and Y_0.

Figure S1 shows the estimated magnetic field amplitude, H_{zpp}, namely, the effective peak to peak value of the magnetic field (H_{max} - H_{min}), see Annex, as a function of the amplitude of vibration of the vibrating surface. Again the linear trend for low Y_0 is observed, justifying the linear response of the GMI sensor under the vibration of the harvester. Note that for higher frequencies, similar slopes are obtained and the linear zone (depicted in the figure) extends to wider amplitudes.
